# Serum levels of CaMKII in patients with hyperventilation syndrome and its correlation with anxiety and depression

**DOI:** 10.1097/MD.0000000000045626

**Published:** 2026-05-12

**Authors:** Liu Luo, Xincheng Mao, Weidong Fu, Xi Song, Liufang Zhou

**Affiliations:** a Department of Anesthesiology, Zhuzhou Hospital Affiliated to Xiangya School of Medicine, Central South University, Zhuzhou, Hunan, China.

**Keywords:** anxiety, CaMKII, depression, hyperventilation syndrome, risk factors

## Abstract

This research aimed to explore the serum levels of calcium/calmodulin-dependent protein kinase II (CaMKII) in hyperventilation syndrome (HVS) patients and its correlation with psychological disorders. This prospective observational study enrolled 168 HVS patients who came to our hospital from February 2021 to January 2023. The serum CaMKII, 5-hydroxytryptamine (5-HT), and brain-derived neurotrophic factor (BDNF) levels were measured by enzyme-linked immunosorbent assay method. Hamilton depression rating scale was used to assess the depression status of all study subjects. Hamilton anxiety rating scale, the self-rating anxiety scale, and the self-rating depression scale were used to further evaluate the psychological status of all patients. The self-rating depression scale, Hamilton anxiety rating scale, and self-rating anxiety scale scores in the depression group were significantly elevated compared to the non-depression group (*P* < .05). The serum CaMKII, 5-HT, and BDNF levels were significantly declined in the depression group compared to the non-depression group. Pearson analysis showed a positive correlation among CaMKII levels, 5-HT levels, and BDNF levels. Serum levels of CaMKII were associated with the psychological status of HVS patients (depression and anxiety). CaMKII could be used to predict depression in HVS patients. CaMKII was a risk factor for depression in HVS patients. This study showed that the serum CaMKII levels decreased in HVS patients with depression. The serum CaMKII level was correlated with 5-HT, BDNF and could be used to predict depression in HVS patients.

## 1. Introduction

Hyperventilation syndrome (HVS) refers to a physiological and psychological response caused by various reasons.^[1,2]^ HVS can lead to tachycardia, sweating, accelerated breathing, continuous elimination of carbon dioxide, resulting in a decrease in CO_2_ partial pressure, and respiratory alkalosis.^[3]^ Common symptoms of HVS include difficulty breathing, anxiety, chest tightness, numbness in the limbs, dizziness, and even convulsions,^[4–6]^ and the occurrence of these symptoms may be related to abnormalities in the respiratory control system, and the loss of stability in autonomous respiratory regulation.^[7]^

Numerous studies have reported a significant association between HVS and psychological disorders, particularly anxiety and depression.^[8,9]^ For example, in a cross-sectional study of 63 severe asthmatic patients, Dafauce et al found that patients with HVS had significantly higher scores on the hospital anxiety and depression scale compared to patients without HVS.^[10]^ All of these findings suggest that investigating the psychological status of patients with HVS may help in developing more effective treatment strategies for HVS. Calcium/calmodulin-dependent protein kinase II (CaMKII) is an important enzymatic molecule present widely in animal cells that affects the functioning of neurons and cardiac cells by regulating calcium ion concentrations.^[11,12]^ A recent study has shown that in a mouse model of depression, both the expression of CaMKII and its phosphorylation were significantly reduced.^[13]^ However, no clinical studies have focused on the serum levels of CaMKII in HVS patients and its relationship with patients’ depressive conditions.

In the present prospective observational cohort research, we aimed to explore the serum levels of CaMKII in HVS patients and its correlation with psychological disorders. This study might reveal the clinical significance of CaMKII in HVS patients, as well as develop more targeted therapies for patients with HVS and psychological comorbidities.

## 2. Methods

### 2.1. Subjects

This prospective observational study enrolled 168 HVS patients who came to our hospital from February 2021 to January 2023. The diagnosis of HVS refers to previous research,^[14,15]^ which can be summarized as follows: patients with symptoms of hyperventilation and a blood gas analysis showing hypercapnia (pCO_2_) < 35 mm Hg; patients with severe respiratory distress, with a Nijmegen symptomology questionnaire score > 23; subjects who received a specific state evaluation, defined as at least 1 positive skin prick test.^[16,17]^ All patients were over 18 years old, and exclusion criteria were as follows: patients with a history of obvious respiratory system disease or other organic diseases causing respiratory distress; patients who had been taking antidepressants, steroids, or hormone drugs for a long time; patients with severe infections or severe liver, kidney, cardiovascular dysfunction; patients with a history of mental retardation or psychiatric disorders. All participants signed a written informed consent. The study was approved by the Ethics Committee of the author’s hospital.

### 2.2. Sample size calculation

*Z*^2^ * *P*(1 - *P*)/*d*^2^ was used to calculate the sample size.^[18]^

Where n is the estimated sample size, *Z* is the standard normal deviate corresponding to a 2-sided confidence level of 95% (*Z* = 1.96), *P* is the estimated proportion of HVS patients with depression, and *d* is the allowable margin of error. Based on previous clinical experience in our institution, approximately 35% of HVS patients present with depressive symptoms (*P* = .35). To ensure a reliable estimation, the margin of error was set at *d* = 0.08. Substituting these values into the formula yielded a minimum required sample size of 168 participants.

### 2.3. Blood sampling measurement

The serum CaMKII, 5-hydroxytryptamine (5-HT) and brain-derived neurotrophic factor (BDNF) levels were measured by enzyme-linked immunosorbent assay method. Briefly, fasting cubital venous blood (5 mL) of all patients was collected. The blood samples were collected and centrifuged at 2000*g* for 15 minutes. After centrifugation, the levels of CaMKII, 5-HT, and BDNF were tested using commercially available enzyme-linked immunosorbent assay kits (CaMKII MBS7253995 MyBioSource, 5-HT MBS701527 MyBioSource, BDNF MBS137923 MyBioSource) strictly according to the manufacturer’s instructions.

### 2.4. Data collection and depression assessment

Demographic and clinical statistics including age, BMI, sex, diastolic blood pressure (DBP), systolic blood pressure (SBP), comorbidities (hypertension, diabetes, and coronary heart disease), etc were collected. A routine whole blood test was performed using an automatic biochemical analyzer (Hitachi 7600, Hitachi Corporation, Japan), and the levels of neutrophil elastase (NE), lymphocyte (LY) and the neutrophil-to-lymphocyte ratio (NLR) were recorded. The partial pressure of carbon dioxide (PCO_2_) level of all patients was measured by a blood gas analyzer (ABL800 FLEX, Radiometer, USA).

Subsequently, we used the Hamilton Depression Rating Scale (HAMD)^[19]^ to assess the depression status of all study subjects. HAMD score of ≥8 was defined as depression. In addition, we also used the Hamilton Anxiety Rating Scale (HAMA),^[20]^ the Self-rating Anxiety Scale (SAS),^[21]^ and the Self-rating Depression Scale (SDS)^[22]^ to further evaluate the psychological status of all patients.

### 2.5. Statistical analysis

The normal distribution of data was confirmed by Kolmogorov–Smirnov analysis. Normal distribution data were expressed by mean ± SD while non-normal distribution data median (range). Mann–Whitney test or Student *t* test was used for comparison between 2 groups. Chi square test was used for rates. Spearman rank correlation was used for correlation analysis. The ROC curve was used to evaluate the predictive value of CaMKII for depression in HVS patients. Logistic regression was performed for risk factors of depression in HVS patients. *P* < .05 regarded a significant difference. All data used SPSS 26.0 for statistical analyses.

## 3. Results

### 3.1. Clinical characteristics of all participants

This prospective observational cohort study recruited 168 HVS patients who were divided into 2 groups based on their HAMD scores: the depression group (HAMD ≥ 8) and the non-depression group (HAMD < 8). The basic information was shown in Table 1. Compared to the demographic and clinical data of the 2 groups, we found that the SDS, HAMA, and SAS scores in the depression group were significantly elevated compared to the non-depression group (*P* < .05). No significant differences were found in age, sex, BMI, SBP, DBP, comorbidities, NE, LY, NLR, and PCO_2_ between the depression and non-depression groups. These findings indicate that the observed differences in psychological status were not influenced by these demographic or clinical factors, highlighting the potential role of CaMKII, 5-HT, and BDNF in mediating depressive symptoms in HVS patients.

**Table 1:** Demographic and clinical data of all subjects.

Variable	HAMD ≥ 8, n = 55	HAMD < 8, n = 113	*P*
Age, yr	43 (20–71)	47 (18–68)	.947
Sex, female (%)	30 (54.5)	53 (47.0)	.325
BMI	25.32 ± 2.09	25.08 ± 2.37	.533
SBP (mm Hg)	123.4615.34	122.06 ± 13.57	.550
DBP (mm Hg)	78.97 ± 8.35	77.81 ± 8.32	.396
NE (10^9^/L)	4.27 ± 0.94	4.30 ± 0.95	.921
LY (10^9^/L)	1.89 ± 0.48	1.87 ± 0.50	.868
NLR	2.22 (1.03–4.61)	2.28 (0.94–6.23)	.762
PCO_2_ (mm Hg)	21.24 ± 5.19	20.45 ± 5.11	.350
HAMD	14 (8–29)	3 (0–7)	<.001
HAMA	12 (4–19)	7 (0–13)	<.001
SAS	43.89 ± 20.34	27.51 ± 15.91	<.001
SDS	55.38 ± 10.31	27.18 ± 14.43	<.001

BMI = body mass index, DBP = diastolic blood pressure, LY = lymphocyte, NE = neutrophil elastase, NLR = neutrophil-to-lymphocyte ratio, PCO_2_ = partial pressure of carbon dioxide, SBP = systolic blood pressure.

### 3.2. The serum CaMKII, 5-HT, and BDNF levels in HVS patients

**Figure 1.**
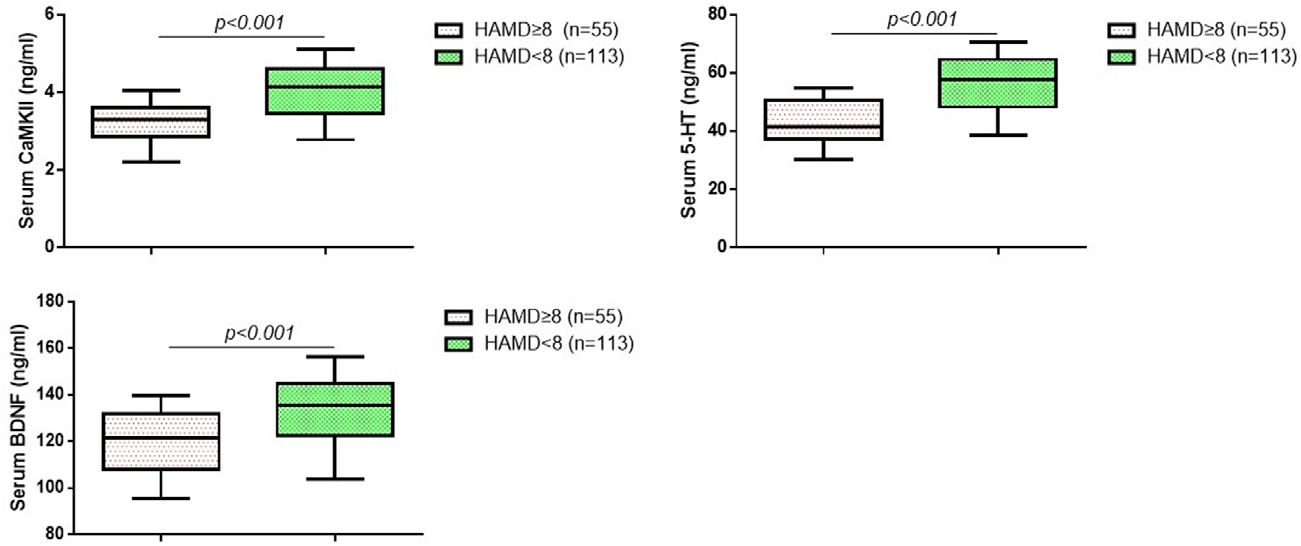
Comparison of serum CaMKII, 5-HT, and BDNF levels between depression and non-depression groups. In boxplots, data were expressed by median (minimum to maximum). 5-HT = 5-hydroxytryptamine, BDNF = brain-derived neurotrophic factor, CaMKII = calcium/calmodulin-dependent protein kinase II.

To further investigate the relationship between serum CaMKII, 5-HT, BDNF levels, and depressive conditions in HVS patients, we draw box plots of all subjects to show the difference in this study. Compared to the non-depression group, the serum CaMKII, 5-HT, and BDNF levels were significantly lower in the depression group (*P* < .05, Fig. 1). These findings suggest that reduced levels of these biomarkers are associated with depressive symptoms in HVS patients, potentially indicating their role in the underlying pathophysiology of depression.

### 3.3. Correlation of CaMKII levels, other biomarkers levels, and clinical data in HVS patients

To investigate the correlation among serum CaMKII, 5-HT, and BDNF levels, as well as clinical data in HVS patients, we performed Spearman rank correlation analysis. Spearman analysis showed a positive correlation among CaMKII levels, 5-HT levels, and BDNF levels (Table 2). Besides, as shown in Table 3, no correlation was found between CaMKII levels and age, BMI, SBP, DBP, NE, LY, NLR, PCO_2_, and SAS. However, the CaMKII levels were negatively correlated with the HAMD scores (*P* < .001), SDS scores (*P* < .001) and HAMA scores (*P* < .001). These results suggested that serum levels of CaMKII were associated with the psychological status of HVS patients (depression and anxiety).

**Table 2:** Correlation analysis among CaMKII and other biomarkers.

		CaMKII	5-HT	BDNF
CaMKII	Pearson correlation	1	0.361	0.236
	*P*		<.001	.002
5-HT	Pearson correlation	0.361	1	0.300
	*P*	<.001		<.001
BDNF	Pearson correlation	0.236	0.300	1
	*P*	.002	<.001	

5-HT = 5-hydroxytryptamine, BDNF = brain-derived neurotrophic factor, CaMKII = calcium/calmodulin-dependent protein kinase II.

**Table 3:** Correlation analysis among CaMKII and other clinical factors.

Variables	CaMKII	
Age	Spearman correlation	0.093
	*P*	.232
BMI	Spearman correlation	-0.114
	*P*	.142
SBP	Spearman correlation	0.004
	*P*	.961
DBP	Pearson correlation	0.010
	*P*	.893
NE	Spearman correlation	-0.043
	*P*	.576
LY	Spearman correlation	-0.016
	*P*	.837
NLR	Spearman correlation	-0.031
	*P*	.694
PCO_2_	Spearman correlation	-0.081
	*P*	.296
HAMA	Spearman correlation	-0.042
	*P*	<.001
HAMD	Spearman correlation	-0.298
	*P*	<.001
SAS	Spearman correlation	-0.095
	*P*	.220
SDS	Spearman correlation	-0.452
	*P*	<.001

BMI = body mass index, CaMKII = calcium/calmodulin-dependent protein kinase II, DBP = diastolic blood pressure, HAMA = Hamilton anxiety rating scale, HAMD = Hamilton depression rating scale, LY = lymphocyte, NE = neutrophil elastase, NLR = neutrophil-to-lymphocyte ratio, PCO_2_ = partial pressure of carbon dioxide, SAS = self-rating anxiety scale, SBP = systolic blood pressure, SDS = self-rating depression scale.

### 3.4. Predictive value of CaMKII for depression in HVS patients

**Figure 2.**
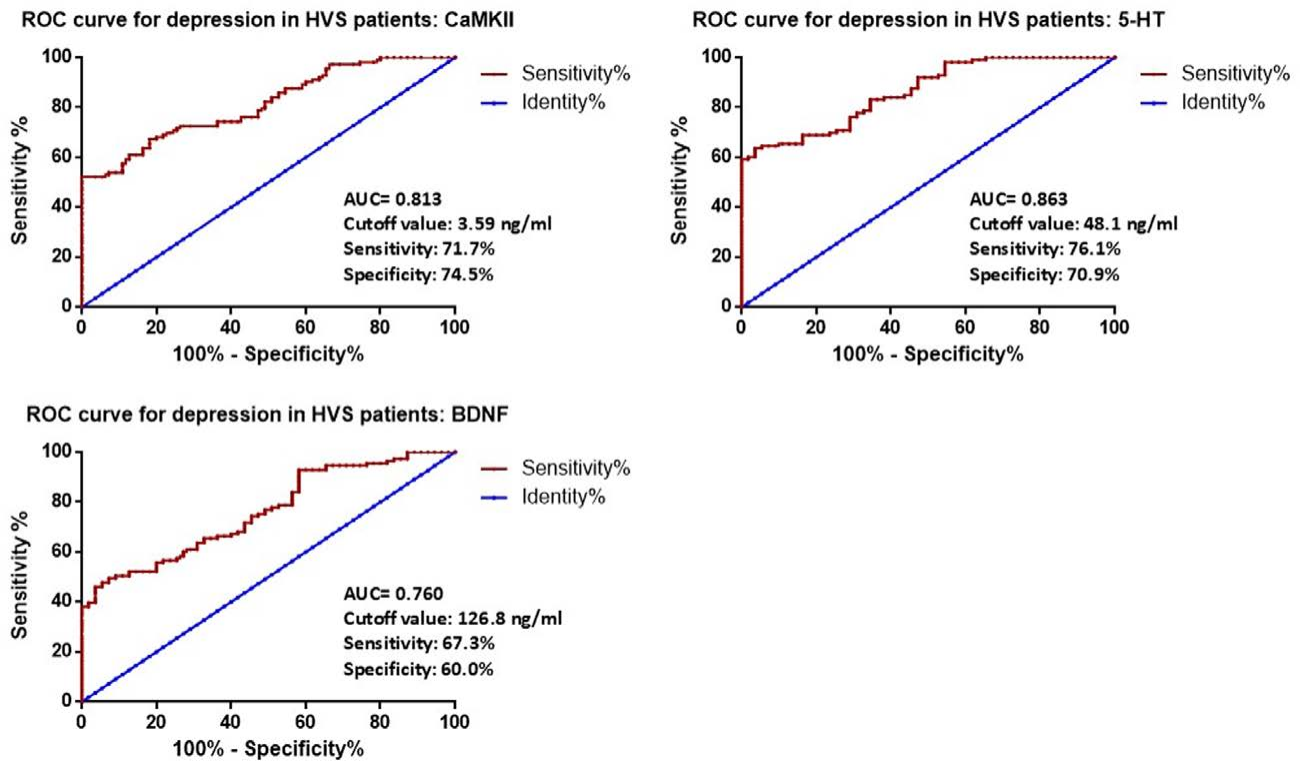
ROC curve for the predictive value of serum CaMKII levels for depression in HVS patients. CaMKII = calcium/calmodulin-dependent protein kinase II, HVS = hyperventilation syndrome, ROC = receiver operating characteristic.

We draw ROC curves to evaluate the predictive value of CaMKII for depression in HVS patients. The results showed that CaMKII could be used to predict depression in HVS patients (Fig. 2), with an AUC of 0.813 (95% CI: 0.750–0.876). The optimal cutoff value was 3.59 ng/mL, yielding a sensitivity of 71.7% and a specificity of 74.5%. At this cutoff, the positive predictive value was 57.4% (95% CI: 45.5–68.4%), and the negative predictive value was 84.0% (95% CI: 75.6–89.9%).

### 3.5. Logistic regression for risk factors of depression in HVS patients

Finally, we used the entry method for logistic regression to analyze the risk factors of depression in HVS patients. For logistic regression, we used 2 models using the entry method, including model 1 (Age, BMI, Sex, SBP, DBP, comorbidities, NE, LY, NLR, and PCO_2_), model 2 (CaMKII, 5-HT, BDNF, SAS, and HAMA). It was found that CaMKII was a risk factor for depression in HVS patients (Table 4).

**Table 4:** Logistic regression for risk factors of depression in HVS patients.

Variables	Wald	Odds ratio	95% CI	*P*
Age	<0.001	1.000	0.973–1.027	.988
Sex	0.726	1.426	0.630–3.227	.394
BMI	0.578	0.936	0.790–1.110	.447
SBP	0.078	0.996	0.965–1.027	.780
DBP	0.361	0.986	0.940–1.033	.548
NE	0.082	1.164	0.410–3.303	.775
LY	0.049	0.761	0.067–8.598	.825
NLR	0.015	0.904	0.176–4.653	.904
PCO_2_	1.611	0.949	0.876–1.029	.204
HAMA	18.986	0.814	0.742–0.893	<.001
SAS	17.834	0.950	0.927–0.973	<.001
CaMKII	19.043	8.847	3.323–23.554	<.001
5-HT	21.819	1.182	1.102–1.267	<.001
BDNF	8.443	1.061	1.019–1.104	.004

5-HT = 5-hydroxytryptamine, BDNF = brain-derived neurotrophic factor, CaMKII = calcium/calmodulin-dependent protein kinase II, DBP = diastolic blood pressure, HVS = hyperventilation syndrome, LY = lymphocyte, NE = neutrophil elastase, NLR = neutrophil-to-lymphocyte ratio, PCO_2_ = partial pressure of carbon dioxide, SBP = systolic blood pressure.

## 4. Discussion

HVS can restrict patients’ daily lives, affect their quality of life, and repeated HVS symptoms may lead to the deterioration of their mental health.^[23]^ Therefore, it is urgent to gain a deep understanding of the psychological disorders among HVS patients and develop personalized intervention methods for their treatment. In our study, we found that the serum levels of CaMKII significantly declined in HVS patients with depression and were correlated with HAMD, SDS, and HAMA scores.

The diagnostic category of HVS is still unclear, and patients may have emotional and psychological issues.^[24]^ Studies have found that adolescents with HVS as the main complaint have poorer social and psychological adaptation when encountering negative life events.^[25]^ Another study indicated that HVS may be associated with anxiety, distressful events, or depressive symptoms.^[26]^ Additionally, HVS patients often struggle to cope with the stress brought about by daily life or work.^[27]^ Several mechanisms have been proposed to explain the relationship between HVS and psychological factors. It has been suggested that anxiety and depression may cause HVS through increased levels of stress hormones, which can lead to hyperventilation.^[28]^ In addition, psychological factors may exacerbate HVS symptoms through increased awareness of breathing patterns and negative thoughts about the symptoms.^[29]^ Therefore, in our study, we grouped all HVS patients according to HAMD scores and detected the levels of 2 depression-related serum markers, 5-HT and BDNF, in both patient groups. Results showed that the serum BDNF and 5-HT levels of HVS patients in the depression group were significantly decreased. These results were also similar to the results of Carniel et al^[30]^ and Ochi et al.^[31]^

There is substantial evidence that CaMKII phosphorylation participates in depressive-like behaviors by activating downstream factor CREB via phosphorylation. In human studies, reduced CaMKII activity has been associated with decreased synaptic plasticity, which is a key factor in the development of depression. Additionally, CaMKII may regulate neurotransmitter systems, including serotonin, that are crucial for mood stabilization, suggesting a multi-faceted role in the pathogenesis of depressive symptoms in HVS patients.^[32,33]^ Therefore, we measured levels of CaMKII in the serum of all patients. Other animal studies are also exploring the relationship between CaMKII and depression. Abdoulaye et al found that the expression of β-CaMKII was significantly increased after Chlorimipramine treatment in an animal model of stroke-induced depression.^[34]^ He et al found that activation of CaMKII expression in neurons can alleviate cognitive dysfunction induced by inflammation in an inflammatory model.^[35]^ Zhang et al confirmed that activation of the CaMKII-related signaling pathway can improve depression-like behavior in rats by enhancing synaptic plasticity.^[36]^ These study results suggest that CaMKII may be involved in the development of depression. However, no clinical studies have focused on the association between CaMKII and depression. Our observational study found that serum levels of CaMKII significantly declined in HVS patients with depression and related to the HAMD, SDS, and HAMA scores. Furthermore, the results of ROC curves showed that CaMKII could be used to predict depression in HVS patients.

This study has several limitations. First, the small sample size may limit the generalizability of the findings. Second, the lack of a control group comprising patients with depression but without HVS restricts the ability to draw definitive conclusions about the specificity of the observed associations. Additionally, potential confounding factors, such as lifestyle differences and genetic predispositions, were not fully accounted for, which may influence the results.

## 5. Conclusion

This study showed that the serum CaMKII levels decreased in HVS patients with depression. The serum CaMKII level was correlated with 5-HT, BDNF and could be used to predict depression in HVS patients. This study might provide a novel targeted therapies for patients with HVS and depression comorbidities.

## Author contributions

**Conceptualization**: Liu Luo.

**Data curation**: Liu Luo, Weidong Fu.

**Formal analysis**: Xi Song.

**Funding acquisition**: Xincheng Mao.

**Methodology**: Weidong Fu.

**Project administration**: Xincheng Mao.

**Software**: Xi Song, Liufang Zhou.

**Supervision**: Liu Luo.

**Visualization**: Liufang Zhou.

**Writing – original draft**: Liu Luo.

**Writing – review & editing**: Weidong Fu, Xincheng Mao.
